# The role of transperineal ultrasound in the evaluation of stress urinary incontinence cases

**DOI:** 10.1590/S1677-5538.IBJU.2020.1100

**Published:** 2021-09-10

**Authors:** Alper Turkoglu, Ayse Deniz Erturk Coskun, Sevcan Arzu Arinkan, Fisun Vural

**Affiliations:** 1 Basaksehir Cam and Sakura City Hospital Department of Obstetrics and Gynecology Istanbul Turkey Department of Obstetrics and Gynecology, Basaksehir Cam and Sakura City Hospital, Istanbul, Turkey; 2 University of Health Sciences Haydarpasa Numune Training and Research Hospital Department of Obstetrics and Gynecology Istanbul Turkey Department of Obstetrics and Gynecology, University of Health Sciences, Haydarpasa Numune Training and Research Hospital, Istanbul, Turkey

**Keywords:** Urinary Incontinence, Stress, Ultrasonography, Urinary Bladder Neck Obstruction

## Abstract

**Purpose:**

To evaluate the use of transperineal ultrasonography while diagnosing stress urinary incontinence (SUI) by comparing the urethral angle (α), posterior urethrovesical angle (β), and bladder neck descent (BND) during rest and Valsalva maneuver in continent women and women with SUI.

**Materials and methods:**

This prospective observational study was conducted with 50 women with SUI and 50 continent women. Transperineal ultrasonography was performed at rest and during Valsalva maneuver. Q-tip test was performed.

**Results:**

During the Valsalva maneuver, both α and β angles were significantly higher in women with SUI (p <0.001). The difference between Valsalva and rest measurements of α and β angles (R α, R β) were also significantly higher in women with SUI (p <0.001). The cut-off point determined for the R α in the diagnosis of stress incontinence was 16° (80% sensitivity, 98% specificity). A statistically significant strong correlation was found between Q-tip test angle and R α value (p=0.000; r=0.890). Q-tip VAS pain scores were significantly higher than ultrasonography VAS pain scores (p <0.001). In relation to the bladder neck descent comparison between the two groups showed that BND was significantly higher in SUI group (p <0.001). The cut-off point determined for BND in the diagnosis of SUI was >11mm (90% sensitivity, 98% specificity).

**Conclusion:**

Transperineal ultrasonography is a practical, reliable, non-invasive and comfortable method for evaluation of SUI. It has the advantage of dynamic evaluation during the Valsalva maneuver. Rotation angles and BND have high sensitivity and specificity for detection of SUI. The change in α angle with Valsalva (Rα) can be used as an alternative to Q-tip test.

## INTRODUCTION

Urinary incontinence is a common and disturbing condition among women. Incontinence can impair social life, physical activity, sexual activity thus affecting emotional and psychological well being. The most common type of incontinence among younger women is stress urinary incontinence (SUI). Stress incontinence is defined as the complaint of involuntary loss of urine on effort or physical exertion, sneezing, or coughing in the absence of bladder contraction (1). With an increase in intra-abdominal pressure, bladder pressure exceeds urethral closing pressure and urine leaks. Two mechanisms explain SUI; urethral hypermobility and intrinsic sphincter deficiency. Since, hammock-like musculofascial fascia provides a backboard support for the proximal urethra, poor anatomic support contributes to SUI by prohibiting compression of urethra (2, 3).

Assessment of bladder neck mobility is a part of the evaluation of SUI. The most commonly accepted method is the Q-tip test. Besides, radiographic tests have been used. Transperineal ultrasonography has also been introduced to evaluate the mobility of the bladder neck and proximal urethra. The majority of the prior studies concentrated on the degree of urethral angle (α), posterior urethrovesical angle (β), and bladder neck descent (BND), but there is a limited number of studies about rotation angles (4–14). Moreover, to the best of our knowledge, there are no studies comparing the Q-tip test with transperineal ultrasonography and investigating Visual Analogue Scale (VAS) scores.

We aimed to evaluate the use of transperineal ultrasonography while diagnosing stress urinary incontinence (SUI) by comparing the urethral angle (α), posterior urethrovesical angle (β), and bladder neck descent (BND) during the Valsalva maneuver in continent women and women with stress urinary incontinence. We also aimed to investigate the correlation of rotation angles with the Q-tip test and investigate relative patient comfort.

## MATERIALS AND METHODS

This prospective observational study was conducted with a total of 100 patients, including 50 patients with stress urinary incontinence (Group I) and 50 healthy continent volunteers (Group II). Ethical approval was obtained from Haydarpaşa Numune Ethics Comitee (HNEAH-KAEK 2019/56). The study group was selected from stress incontinence patients scheduled for surgery. The control group was selected from volunteers among outpatient gynecology clinic patients who did not have urinary incontinence complaints and who fulfilled the inclusion criteria.

Patients were asked to fill the validated Turkish Urinary Distress Inventory short form (UDI-6) and Overactive Bladder Questionnaire (OAB-V8) since overactive bladder can frequently accompany SUI (15). Complicated SUI patients (16), pregnant women, puerperants, patients with overactive bladder (OAB-V8 score ≥8), pelvic organ prolapse exceeding the hymen, residual urine more than 150cc or having voiding difficulty questioned in a non-directing open manner (17), urinary tract infection, nocturia, post-coital incontinence, diabetes mellitus, and known neurological disease were excluded. Having had genitourinary surgery, being younger than 18 years of age, having an active infection, malignancy, or gynecological disease and usings drugs affecting continence were also included in the exclusion criteria.

**Figure 1:**
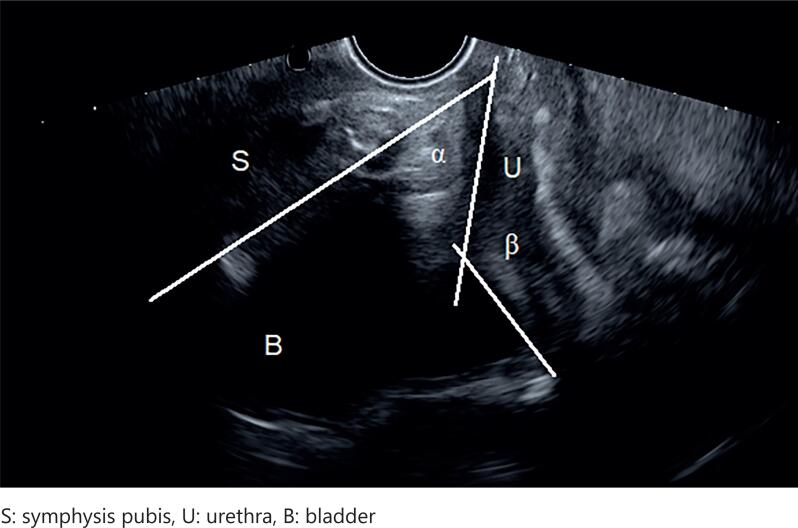
α and β angles at rest (presented as image in the form of TIFF file).

Ultrasonography was performed in the lithotomy position with the MINDRAY DC-7 device. Comfortably filled bladder (150-200cc) was required and checked with ultrasonographic volume formula from measurements obtained transabdominally. Transperineal ultrasonographic measurements were made from the interlabial area by applying the vaginal probe (V 10-4, 6.5 MHz) to the symphysis pubis to obtain the sagittal view described by Dietz et al. (18). The symphysis pubis, bladder, and urethra were visualized. The central axis of the symphysis pubis appearing as an oval, the proximal urethra axis, the posterior bladder wall axis and the horizontal line crossing the posteroinferior margin of the symphysis pubis were marked. The angle between the proximal urethra axis and the long axis of the symphysis pubis (α angle) and the angle between the proximal urethra and the posterior bladder wall (β angle) were measured from the marked lines (Figure-1). Patient was asked to fully strain by verbally directing to push and cough, and the maximum Valsalva image was obtained with the help of cine-loop function, the mentioned lines were drawn with tracers and angles were measured from printouts. The differences of angles at rest and strain (rotation angles) were recorded as R α and R β. For the calculation of bladder neck descent (BND), the distance between the location of the bladder neck and the horizontal axis passing through the distal end of the symphysis was measured on the images taken during rest and Valsalva, the difference was calculated and recorded as BND. All urogynecological evaluations and all ultrasonographic measurements were made by a single observer.

Q-tip tests were performed by a different observer in the standart way and patients were asked to rate their discomfort for the two methods using a 0-10 visual analog scale (VAS). The main outcome measures were the urethra angle (α), posterior urethrovesical angle (β) at rest and Valsalva, R α, R β and bladder neck descent (BND). Secondly, the relationship between rotation angles (R α, R β) and Q-tip test results and the pain scores of the tests performed were investigated.

The sample size was calculated via a simple random sampling method in the studied universe. Informed consent was taken from all patients. Statistical analyses were done by using Statistical Package for the Social Sciences (SPSS; Version 22.0, IBM Turkey). Power analysis was performed (Power:0.80, α:0.05 n:20). Descriptive statistics (mean, standard deviation, frequency) were used. Normality distribution of the data was evaluated with Shapiro Wilks test. Student's t test and Mann-Whitney U tests were used for normally distributed and non-normally distributed variables, respectively. In the comparison of qualitative data, the Fisher Freeman Halton test and Continuity (Yates) Correction were used. Pearson correlation analysis was used to examine the relationships between parameters that show normal distribution. Cut-off points were determined based on the ROC curve analysis. Logistic regression analysis was applied for multivariate analysis. Significance was evaluated at the level of p <0.05 and p <0.001.

## RESULTS

The mean age of the cases included in the study was 46.83±8.32 years. There was no statistically significant difference between study and control groups in terms of age, BMI, parity, educational status, obstructed labor, macrosomic birth history and menopause (p >0.05) (Table-1). The comparison of UDI-6 scores of patients with SUI (5.1±1.5) and controls (2.5±1.8) was statistically significant (p=0.000).

**Table 1:** Comparision of general and ultrasonographic characteristics of patients.

		Group I	Group II	p
		(SUI) (n=50)	(Control) (n=50)	
**General characteristics**				
	Age	46.56±7.82	47.1±8.86	0.747
	BMI (kg/m²)	28.27±5.75	27.28±4.82	0.351
	Parity	2.62±1.63	2.18±1.49	0.273
	Obstructed labour	9 (18%)	3 (6%)	0.124
	Macrosomic labour	8 (16%)	3 (6%)	0.201
	Menopause	7 (14%)	12 (24%)	0.202
	UDI-6	5.1±1.5	2.5±1.8	0.000
**Ultrasonographic measurements**				
	α angle (rest) (°)	62.44±4.75	61.42±6.21	0.358
	α angle (Valsalva) (°)	86.66±11.01	69±7.08	0.000
	β angle (rest) (°)	119.76±7.54	119.18±7.24	0.696
	β angle (Valsalva) (°)	139.62±9.1	125.48±7.15	0.000
	R α (°)	24.42±9.57	7.58±3.23	0.000
	R β (°)	19.76±7.39	6.2±3.24	0.000
	BND (mm)	16.6±4.22	6.53±1.69	0.000

The α and β angles measured at rest were not statistically different between SUI and control groups (p >0.05). After Valsalva, the α angle was significantly higher in SUI group (86.66±11.01) than control Group (69±7.08) (p=0.000). Also, Valsalva β angle was significantly higher in SUI group (139.62±9.1) compared with control group (125.48±7.15) (p=0.000) Regarding the change in angles after Valsalva; R α was significantly higher in SUI group than control group (24.42±9.57 vs. 7.58±3.23) (p=0.000). R β was also higher in the SUI group (p=0.000). Comparing the BND between the two groups it was significantly higher in SUI Group (16.6±4.22 vs.6.53±1.69) (p=0.000) (Table-1).

Ultrasonographic measurements were significantly correlated with Q-tip test angles: α Valsalva (r:0.809 p: 0.000), β Valsalva (r:0.0.658 p:0.000), R (r:0.890 p:0.000), R β (r:0.773 p:0.000), BND (0.925 p: 0.000). Ultrasonographic measurements were significantly correlated with SUI: α Valsalva (r:0.699 p:0.000), β Valsalva (r:0.659 p:0.000), R α (r:0.771 p:0.000), R β (r:0.760 p:0.000), BND (r:0.847 p:0.000). Q-tip test VAS pain scores (4.20±1.14) were significantly higher than ultrasonography VAS pain scores (2.06±0,95) (p=0.000; p <0.05).

Logistic regression analysis was performed to predict SUI with urethral angles (α Valsalva, β Valsalva, R α and R β). Model was found statistically significant (p=0.001, p <0.05), Negelkerke R square was found as 0.919 and the overall coefficients of the explanatory variables were high (95%) (R α (OR: 2.144, CI:1.043-4.408), R β (OR:1.293,CI: 0.974-1.717), α Valsalva (OR: 0.791, CI: 0.55-1.137), β Valsalva (OR:1.234, CI: 0.979-1.554). Among urethral angles R alpha was the significant factor related to SUI (p=0.038). If we added both R alpha (OR:1.4 p:0.106) and BND (OR:2.7 p:0.005) to regression model, BND was the significant factor related to SUI. These results showed that BND was the independent predictor of SUI, while R alpha was the most important measurent among urethral angles.

**Figure 2:**
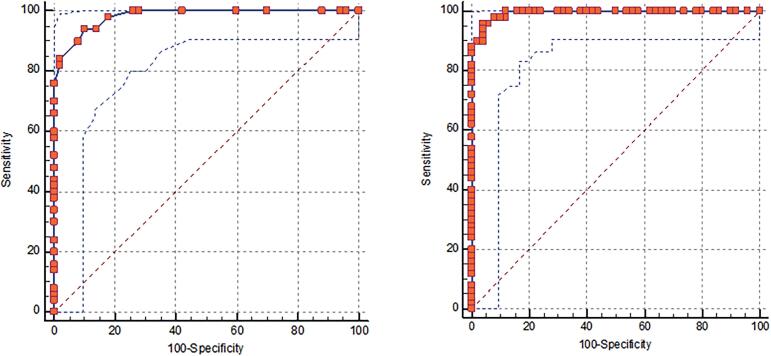
The two graphics can be labeled as a and b for clarity. and the legend can be changed as: ROC analysis of R α (a) and BND (b) for SUI.

The cut-off point determined for the R α in the diagnosis of stress incontinence is >16°. The sensitivity of this value was found to be 80% and specificity 98%. The ROC analysis results for R α are shown in Figure-2A (AUC:0.982, CI:0.963-1, p=0.000). The cut-off point determined for BND in the diagnosis of SUI was >11.2mm (90% sensitivity,98% specificity)(p=0.000) (Figure-2B). When all ultrasonographic measurements were compared, BND was found to have the highest sensitivity and specificity.

## DISCUSSION

Accurate evaluation of the stress incontinence is of great importance for preventing unnecessary medical-surgical interventions. Questionnaires can be used to assess patient's symptoms. International consultation on incontinence modular questionnaire - female lower urinary tract symptoms (ICIQ-FLUTS) can be used for this purpose. We used UDI-6 and OAB-V8 since ICIQ-FLUTS was not validated in Turkish at the time of our study. It has been validated in both Turkish and Brazilian Portuguese in 2020 (19, 20). Q-tip test, radiographic techniques, and ultrasonography have been used for the evaluation of SUI. Use of urodynamic studies are controversial, especially in uncomplicated SUI cases (21). A reliable, non-invasive, and cost-effective method is required for the evaluation of SUI cases. In this study, bladder and urethra angles and displacement dynamics during the Valsalva maneuver were analyzed in patients with and without SUI on transperineal ultrasonography. With hypermobile urethra which is considered an underlying pathophysiological reason for SUI the proximal urethra will rotate more postero-inferiorly and the bladder neck will move lower during Valsalva. The longitudinal axis of the symphysis pubis bone is used as a reference line since it does not move during Valsalva for α angle measurement, and a horizontal line crossing the postero-inferior margin of the symphysis pubis is used to observe the descent of the bladder neck during Valsalva. Bladder neck descent, symphysis pubis-urethra angle (α angle), posterior urethrovesical angle (β angle) are the commonly measured parameters in the literature. However probe localization, bladder fullness, and the intensity of the Valsalva maneuver were not standardized, resulting in no consensus about the cut-off values for SUI differential diagnosis.

Dietz et al. showed that the full bladder is less mobile than the empty organ leading to less BND and smaller rotation angles. They emphasized that bladder fullness should be stated in studies (22). In the preliminary measurements we conducted before starting our study, we observed that it was harder to view the angles with an empty bladder as suggested by Pizzoferrato et al. (23). Views were obtained more easily with a full enough bladder that would not restrict the Valsalva maneuver by creating anxiety of urine leakage. We chose bladder volume to be 150-200cc similar to the study by Al-Saadi et al. (8).

Xiao et al. examined the role of transperineal 3D ultrasonography in the evaluation of SUI. The threshold value of BND was determined as 24mm (66.4% sensitivity, 84.5% specificity). The study indicated that transperineal ultrasonography was inadequate in predicting SUI but could reduce unnecessary urodynamics and treatment by identifying cases without SUI (9). Another study by Naranjo-Ortiz et al. concluded that BND >25mm was in favor of urethral hypermobility (58% sensitivity, 60% specificity) (24). In the study by Hajebrahimi et al. BND was recorded as 15.64±9.65mm in the SUI group and 8.13±9.16mm in the control group (p <0.01) (10). Li et al. also examined the use of transperineal ultrasonography for SUI evaluation. BND, α, and β angles were significantly higher in the SUI group compared to the control group. They stated the mean BND value in the SUI group as 2.19 (±0.80) cm and the BND value in the control group as 1.14 (±0.66) cm (p <0.001) (11). In our study, the mean BND value of the SUI group (16.6±4.22mm), the mean BND value of the control group (6.53±1.69mm), and the threshold was determined as 11.2mm. These values are closest to those reported by Hajebrahimi et al. where bladder filling was 200-300cc. similar to our study. Other mentioned studies were done with an empty bladder and BND values and threshold values measured were higher than those of our study. Another reason for this could be that our study was performed in a homogeneous population and that cases with pelvic organ prolapse were not included. In the study by Dietz et al. BND was reported as a predictor of SUI whereas rotation angles were not (25). Overall, as concluded in a recent review BND is easy to perform and reliable to investigate urethral mobility (26).

Considering the differences of angles in SUI, Al-Saadi et al. evaluated the α and β angles during rest and Valsalva maneuver. Similar to our study, bladder filling was 150cc. The mean α angle (rest) was significantly higher in the SUI group (64.37 vs. 43.9) (p <0.01). The mean α angle (Valsalva) was also significantly higher in the SUI group (83.80 vs. 54.43) (p <0.01). Besides, the mean β angle values were significantly higher in the SUI group both at rest and Valsalva (8). Yang et al. similarly reported higher resting and straining angles in the SUI group compared to the controls (14). Sendag et al. reported higher β angles at rest and Valvalva in the SUI group, whereas they reported α angle to be higher only at Valsalva similar to our study (6). In our study, the mean α and β angles at rest were not statistically different between the groups. This could result from our exclusion of POP cases. The demographic characteristics of the groups that may affect resting anatomy such as parity, macrosomic delivery and BMI were not compared in the study by Al-Saadi et al. therefore the difference during rest may be a result of the choice of the control group in that study.

Mean α and β angles measured during the Valsalva maneuver were significantly higher in the SUI group of our study. This is consistent with existing literature, however the numerical values vary, probably due to choice of different methodology. Yang et al. stated that it is not possible to select a threshold value for the angles due to the wide range of overlap between groups in their study, whereas Al-Saadi et al. suggested a threshold of 58.5° for α angle Valsalva with a high sensitivity and specificity (≈97%). The same study suggested that the difference between Valsalva and resting α and β angles was significantly higher in the SUI group (R α SUI 19.43 vs. control 10.53, p <0.001; and R β SUI 28.30 vs control 16.33, p <0.001) (8, 14). Similarly, in our study, R α and R β values were significantly higher in the SUI group.

To our knowledge, there are no studies evaluating the correlation between angles measured on transperineal ultrasonography and Q-tip test angle. In our study, the correlation of the Q-tip test with the R α angle was 89%, and the correlation of the Q-tip test with the R β angle was 77.3%. Anatomically, the R α angle, which evaluates the proximal urethra rotation with respect to the fixed line symphysis axis, is expected to be compatible with the Q-tip test angle. In all participating women, Q-tip test VAS pain score was higher than transperineal ultrasonography (4.20±1.14, 2.06±0.95, respectively). Meyer et al. suggested Valsalva with a cotton swab inserted into the vagina as an alternative to the Q-tip test. The comparison of the VAS scores showed that the patients found the vaginal stick method to be less uncomfortable (27). In clinics where ultrasonography is part of the examination, urethral hypermobility can be easily observed and measured with the change in α angle with Valsalva (R α) instead of the rather uncomfortable Q-tip test. Larger studies including patients with different degrees of prolapse are needed to determine a cut off value.

The limitations of this study include firstly, lack of urodynamic proof for SUI. SUI patients were all surgery candidates with observed SUI with cough test. We were strict about not including patients with pelvic organ prolapse to both groups. We also excluded complicated SUI patients who require urodynamic study according to ACOG guideline such as those with prior pelvic surgery, urgency, postvoid residual volume >150cc, Q-tip test <30 (16). Another limitation is the lack of standardization of the Valsalva maneuver, which is the case in majority of the studies since measurement of intra-abdominal pressure is not easily done and can require rectal probes. Thirdly, we did not investigate Levator Ani Muscle (LAM) injury which is better observed with 3 dimentional ultrasonography. LAM injury is important in the pathogenesis of bladder neck mobility (28). Another limitation is our usage of the vaginal probe instead of the convex probe since majority of the related literature used the latter. We chose to use the vaginal probe with curved array tip since we observed the exact same anatomical structures in the same plane with a clearer view.

## CONCLUSION

Resting bladder α and β angles are similar in cases without POP. α and β angles and rotation angles increase significantly with Valsalva maneuver in patients with stress incontinence. BND measurement and R α measurement are very reliable to predict Q-tip test and SUI. Patients report lower VAS scores for ultrasonography compared with the Q-tip test. Ultrasonography is a reliable, non-invasive and comfortable method that provides dynamic evaluation in the examination of urethral mobility and can substitute Q-tip test where applicable.
